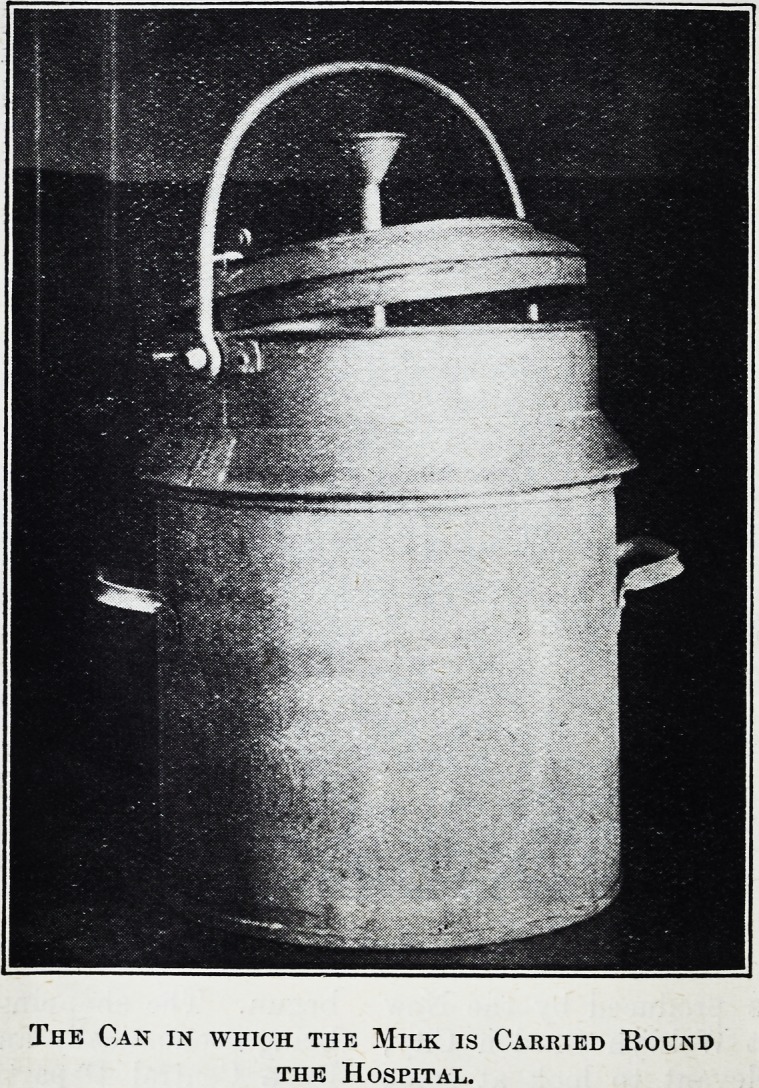# Clean Milk for Consumptives: Hospital Milk from a Garden City

**Published:** 1924-01

**Authors:** 


### CLEAN MILK FOR CONSUMPTIVES.

### HOSPITAL MILK FROM A GARDEN CITY.

WHEN we were invited to go down to Welwyn Garden City and see the conditions under which the milk is produced which is consumed at the City of London Hospital for Diseases of the Heart and Lungs, and afterwards to visit the hospital —invitations to which we gladly responded—it was probably not in the mind of Mr. George Watts, the courteous Hospital Secretary, that our visits would leave us reflecting on the saddening study of contrasts in environment which they afforded. It is our main purpose to write of milk as produced by the New Town Agricultural Guild at Welwyn Garden City, but it is by no means irrelevant to look at some of the more general objects of the New Town movement, particularly in regard to agriculture.

### New Town Aims.

On four square miles of beautiful Hertfordshire country land there is growing daily a pioneer town in which seventeen hundred people are already settled, where it is sought to work out in practice some of the theories which are set forth in the book, "New Town: a Proposal in Agricultural, Industrial, Educational, Civic and Social Reconstruction," from which we make one or two extracts. The promoters :—

Seek to develop a new type of town in which the townsman is not altogether ignorant of the life of a rural community, and a new type of rural worker who knows something of corporate life and shares some of the educational, social, intellectual and spiritual advantages of a town. These are best realised in the association of men and women in work and in play, in manufacture, in music, in drama and in worship. Thus we hope to do something to help to colonise England, to show that her broad acres are not worked out, that they can support a vigorous population and hundreds of healthy towns, and that her people will thrive most, and be healthiest and happiest, when the keynote of all their efforts is association in work and in the rewards of work.

### The Awakening Farm Worker.

In pursuit of these aims a good beginning has been made. Some 600 houses have already been erected in the Garden City, and every week sees new buildings begun. The shopping needs of the community are being economically and very efficiently provided for by a Central Department Store controlled by the Garden City Company. There are already eighteen departments with a weekly turnover of £700. It is, however, with the agricultural enterprise within this growing community that we are concerned. Let us quote again from the book "New Town" as to the underlying idea :—

We picture in our main farming enterprise a company of men, who would otherwise be small-holders, farmers, bailiffs or labourers, joined together to control and make the best use of the New Town farm-lands. There will be those who have been brought up as labourers on the land, skilled men, be it remembered, masters of many operations, and cognisant of many of Nature's secrets. In practice to-day the farm labourer, on some farms, does help his master with advice and opinion, although he may not share in the profits resulting from his advice. We hope to give him a conscious share in the management of the farm, and believe that he will rise to the opportunity. A weekly Committee Meeting at which reports on work done, and plans for future work, are made will bring new interest into the labourer's life. Then, by degrees, we shall be also introducing the new type of towns-

A Room in the Hostel : New Town Agricultural Guild.

man-landworker, who has been educated up to eighteen and will be able to co-operate even more intelligently. And there will be always the skilled organisers and directors of the different departments.

### The Milk Supply.

And then, as to the milk supply :—

The provision of pure milk will be one of the chief concerns of the Farming Company, and, when New Town has its full population, this will probably be its largest department, since it will demand that a considerable part of the farm be devoted to milk production on the most modern intensive system.

### The Agricultural Guild.

These proposals are being put into practice. The New Town Agricultural Guild, a self-governing body of about fifty agricultural workers, is in existence, cultivating close on 1,000 acres, and trying to maintain a high standard of living for the farm labourers, in addition to giving them a large share in the responsibility for the work which they are doing. The governing Committee of the Guild consists of nine members, two of whom are nominated by the New Town Trust, which supplies the working capital, one by the Parish Council, one by the Trade Union concerned, and five by the workers. Mr. Harris Smith, a large farmer in Essex, is Chairman of the Guild, and it is under the direct management of the Director of Agriculture, Mr. Henry Henshaw, with specially chosen heads for the various departments. Mr. Henshaw, before joining the staff of the Guild, had a unique experience as Farm Superintendent and Demonstrator, Cambridge University Agricultural Department for ten years, Manager and Pedigree Live Stock Agent for Chivers and Sons seven years, and Director of Farm Settlement under the Ministry of Agriculture three years. The Guild, as well as carrying on ordinary arable farming, owns a herd of pedigree cattle for the production of "Certified Milk," the highest grade recognised by the Ministry of Agriculture; other cattle for the supply of clean milk; large herds of Middle White and Large White pigs; poultry on a modern and extensive scale; a newly planted orchard of 23 acres; and engages in market gardening, bee-keeping and cultivation under glass. Much of the produce is sold through the Stores in the City itself, thereby eliminating cost of carriage, and it is the object of the Guild as far as possible to supply the needs of the growing city from its own agricultural belt. The Directors have adapted an old farmhouse and are running it as a Hostel for workers. They are fortunate in having so thoroughgoing and enthusiastic a Warden as Mrs. Ralph Crowley. The Hostel is capable of housing 30 residents and occasional guests, and has proved so successful that it may be necessary to arrange for additional accommodation.

The Can in which the Milk is Carried Round the Hospital.

### Clean Milking.

At the Lower Handside Farm we were shown the pure-bred Dairy Shorthorns, some thirty in number, being milked under conditions which it would be difficult to better. The dominant note was cleanliness. Every cow was clean from the nose to the tip of the tail; every day they are thoroughly groomed —brushed, washed, clipped. The floor of the cowshed was spick and span, with clean straw litter thrown down, the shed well lighted and well ventilated. And a most placid and contented herd of cows were being milked by electricity, by the de Laval process. The regular rhythm of the milking is not subject to the interruptions and variations of hand milking, and the milk flows from the teat through rubber tubing into the covered-in can; the tubing itself is regularly sterilised by high steam pressure.

### The Hole in the Wall.

Immediately the cow is milked, the pail is taken to the end of the shed, and the milk is poured through an aperture in the wall into the cooling apparatus in the bottling room. The little cover over the hole is then at once shut. The milk having passed over the cold-water-lined grid which constitutes the cooling apparatus, is received into the bottles kept in readiness by the white-overalled dairy maid, who closes the bottle and fastens on the cap which contains, in accordance with the Ministry of Health requirements, the words: "Certified milk, produced and bottled on Monday (or as the case may be) by New Town Agricultural Guild, Limited, at Handside Farm."

### Certified Milk.

Samples of the certified milk are taken from time to time without previous notice by the Ministry of Health. Their officers take the samples usually from the loaded carts, and subject them to bacteriological tests, the details of which are sent to the producer. Certified milk, so tested before delivery to the consumer, must at no time be found to contain more than 30,000 bacteria per cubic centimetre, or any *bacillus coli* in one-tenth of a cubic centimetre. We were informed that at the last test taken 2,680

bacteria only were counted. Every cow in the herd has passed the Government tuberculin test, and is carefully examined by a veterinary surgeon from time to time, and every animal has to pass the half-yearly tuberculin test to ensure that it is free from tuberculosis. Here, then, at Handside Farm, certified milk was being bottled within a few minutes of leaving the cow and without exposure to the air, except for the fraction of time in which it ran over the cooler. It is the purest milk that can be supplied, and it is available to the residents of Welwyn Garden City at the lowest price at which it can possibly be supplied by those who believe that milk can never be more wholesome or nutritious than it is at the moment it leaves a healthy cow.

### The Milk at the Hospital.

It is satisfactory to find that, consistently with their theories, the New Town Guild can from their farms, primarily worked for the residents, supply the needs of an East-End hospital. Both classes of milk are supplied to the City of London Hospital for Diseases of the Heart and Lungs, the tuberculin-tested milk, just as it comes from the cow, for consumption by the patients, and the other milk mainly for cooking and general hospital purposes. The Hospital authorities congratulate themselves on the fact that each morning at 9 o'clock, at King's Cross Station, they receive milk of that morning's milking. We were not surprised to find that Mr. Watts, the Secretary of the Hospital, and Dr. Gloyne, the pathologist, take great interest in their milk supply. In the first place the milk is carefully stored in cool cellarage. (It comes from the farm in churns which are specially fitted with caps which cannot be tampered with in transit without leaving evidence.) The covered cans in which the milk is carried around the hospital wards are deserving of special mention; they are ice-lined, and the stirring-rod, as shown in the illustration, is fitted to the can, so that it cannot be removed and left lying about on a possibly dusty floor or shelf.

### Independent Tests.

Dr. Gloyne explained to us that separate tests are taken of both classes of milk, so far with very satisfactory results. Bearing in mind that 30,000 bacteria to the cubic centimetre are permissible in certified milk, and that ordinary milk consumed in London frequently contains many millions of bacteria per cubic centimetre, the following figures of bacterial counts made in the six weeks preceding the day of our visit are interesting :—

| | | Tuberculin Tested Milk (corresponding with Certified Milk). | Non tuberculin tested. |
|---|---|---|---|
| October | 15 | 16,000 | 26,000 |
| „ | 30 | 10,700 | 136,000 |
| November | 6 | 47,000 | 84,600 |
| „ | 13 | 25,000 | 20,000 |
| „ | 20 | 7,600 | 6,500 |
| „ | 27 | 4,200 | 2,000 |
| Average | | 18,400 | 45,800 |

It is noteworthy that the ordinary milk gave even better results in the last two counts than the special milk. We were pardonably curious as to how 136,000 bacteria—to say nothing of much larger quantities—are counted, and Dr. Gloyne explained that the milk to be tested is first diluted 1,000 times, and then the colonies of bacteria are counted without difficulty in a circular glass Petrie dish, the results being multiplied by 1,000. Samples of milk are also placed in a special apparatus and swirled round for the settlement of a deposit, injected into guinea-pigs. No tubercle bacillus was found in any such inoculations. The bacillus coli was entirely absent.

### Awaiting "The Day."

As we left the Hospital we reflected that, pending the arrival of the day when our Health Authorities will bestir themselves to stamp out those dreadful environmental conditions which are to be found within a stone's throw of the Hospital, and which do so much to create fresh cases of diseases of the heart and lungs, it is, at any rate, comforting to find that both producers and Hospital authorities are giving such solicitous care to the question of so important an article of diet as the milk which is supplied to the patients.

## Figures and Tables

**Figure f1:**
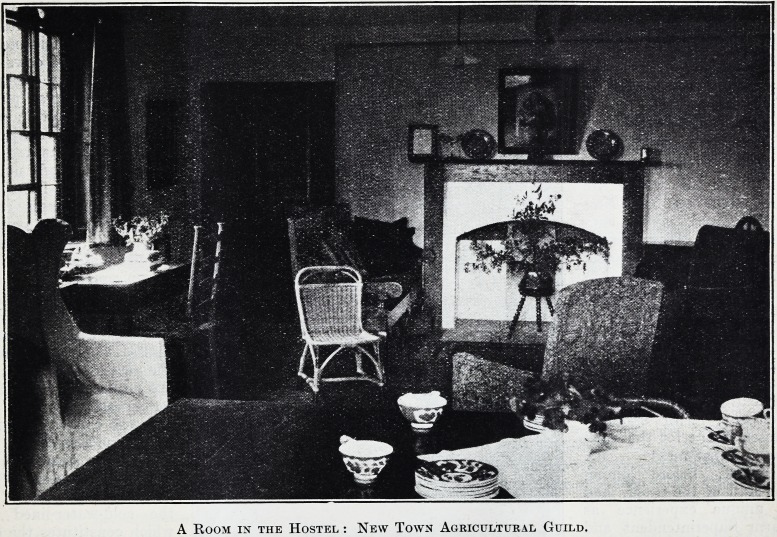


**Figure f2:**